# Enhancing Bacterial Adhesion with Hydro-Softened Chitosan Films

**DOI:** 10.1021/acsmacrolett.5c00374

**Published:** 2025-07-30

**Authors:** Hojin Seo, Xiaoqing Yu, Anuja Tripathi, Julie A. Champion, Tequila A. L. Harris

**Affiliations:** † George W. Woodruff School of Mechanical Engineering, 1372 Georgia Institute of Technology, Atlanta, Georgia 30332-0405, United States; ‡ School of Chemical and Biomolecular Engineering, Georgia Institute of Technology, Atlanta, Georgia 30332-0405, United States

## Abstract

In applications ranging from microbial fuel cells to targeted drug delivery, bacterial adhesion is critical for surface interactions and functional performance. Current strategies for modulating bioadhesive properties of chitosan largely rely on biochemical functionalization – ligand grafting, surface charge manipulation, and polymer blending. Here, we introduce a mechanically driven framework based on hydro-softening – a physical process that modulates adhesion outcomes by tuning elasticity and interfacial energy without introducing foreign chemical species. Hydro-softened chitosan thin films entropically entrap interfacial water during substrate-mediated condensation, forming pseudosolid water domains that lower both the elastic modulus and effective work of adhesion. We integrate changes in these mechanical effects into a Griffith-criterion-derived theoretical adhesion model, coupled to a stochastic simulation incorporating extended Derjaguin–Landau–Verwey–Overbeek (DLVO) interactions. The resulting predictions of enhanced bacterial adhesion were validated experimentally through quantitative Scanning Electron Microscopy (SEM) analysis and morphological classification. Hydro-softened chitosan thin films exhibited over 5-fold greater adhesion compared to unsoftened chitosan thin films, primarily through increased single-cell attachment. These findings demonstrate that substrate mechanics alone can govern quasistatic bacterial attachment in *in vitro* settings. This work establishes hydro-softening as a chemically passive yet effective process-driven strategy for engineering bioadhesive interfaces. It further demonstrates that mechanically induced changes can influence biological interactions even at the cellular scale.

## Graphical abstract

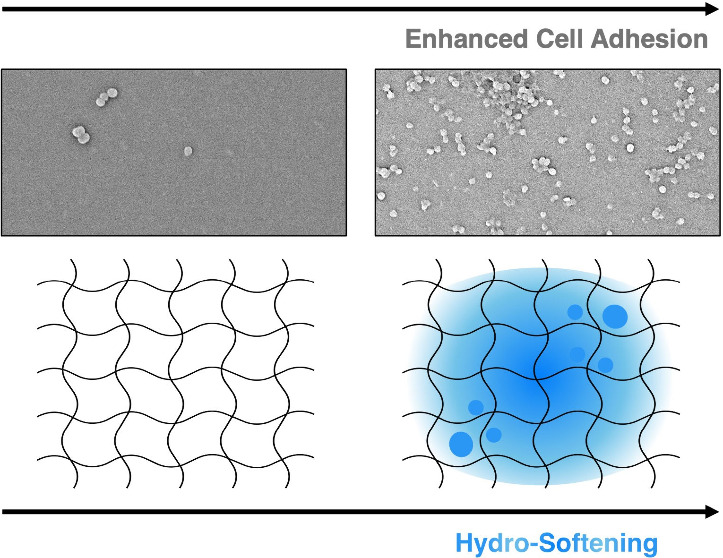

Enhanced cellular and bacterial adhesion is advantageous for many applications, including but not limited to microbioelectrochemical carriers^1–5^ and targeted drug delivery agents.^6–11^ In biosensing systems^12–15^ and microbial fuel cells,^16–19^ for example, enhanced bacterial adhesion can improve the system efficiency by facilitating increased microbial interactions with electrode surfaces. In targeted drug delivery,^20–23^ increased adhesion of therapeutic bacteria can promote localized treatment.

Chitosan is an antibacterial^24–27^ and nonadhesive biomaterial^28–30^ with an intrinsically low surface affinity.^31,32^ Leveraging its biologically active properties, studies have demonstrated modulation in bacterial adhesion with chemical functionalization of chitosan.^33–37^ Chitosan surfaces functionalized with κ-carrageenan multilayers^38^ have been shown to reduce bacterial adhesion by modulating surface charge and hydration. Glycan-functionalized chitosan microspheres,^39^ on the other hand, have been used to enhance selective adhesion of *Helicobacter pylori* through adhesin–glycan interactions. These strategies highlight a single-phase dependency in the two-phase model of bacterial adhesion^40^ (Figure 1), in which quasistatic physicochemical forces mediate initial attachment, while time-dependent long-term adhesion is sustained by molecular binding and biofilm maturation. Here, phase one of the bacterial adhesion process is determined by instantaneous energetic compatibility, governed by surface energy and mechanical properties, whereas phase two of the bacterial adhesion process is regulated by dynamic molecular engagements and hysteresis. With κ-carrageenan-modified chitosan, bacterial adhesion is mitigated mainly through phase one suppression, where altered surface charges limit initial energetic compatibility. With glycan-functionalized chitosan microspheres, enhanced phase two engagement with strain-specific adhesin–glycan interactions enables stable and selective bacterial attachment. Our work builds on this understanding by investigating how hydro-softened chitosan thin films influence phase one compatibility, with implications for mechanical mediation of bacterial engagement.

**Figure 1:**
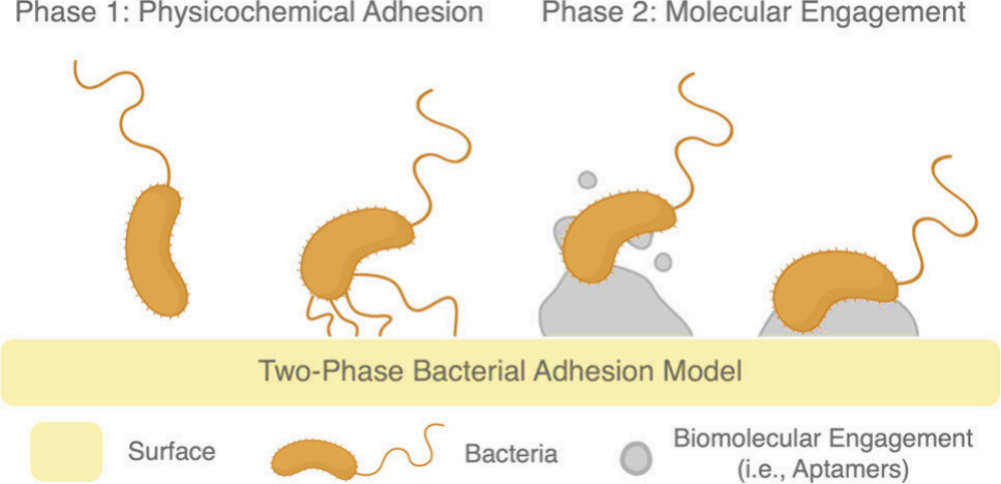
Two-phase model of bacterial adhesion.

While most adhesion studies have focused on biochemical strategies, such as surface functionalization,^41–43^ charge modification,^44–46^ or ligand incorporation,^47–49^ recent work has begun to explore the underlying mechanical processes^50–52^ as means of modulating microbial attachment. While typically observed at the molecular level, mechanisms such as shear-enhanced binding via catch-bonds highlight how mechanical forces can modulate adhesion dynamics.^53^

Here, we introduce hydro-softening of chitosan^54^ as a mechanical, process-driven method to enhance bacterial adhesion as an alternative to chemical modifications. Hydro-softening involves modulating the retention of interface-confined water within the chitosan material matrix to reduce its rigidity without altering its chemical identity. Chitosan is typically rigid due to its highly crystalline β-1,4-linked glucosamine backbone,^55^ which forms extensive intermolecular bonds that reinforce its structural stiffness. With periodic and entropic confinement of localized hydration^54^ within chitosan, however, the elastic modulus is drastically reduced (see SI, Figure S1), placing the material within the range of elastomers, without introducing any chemical modifications. This reduction in stiffness lowers the energetic barrier for bacterial adhesion, thereby enhancing attachment at the interface.

To theoretically and numerically evaluate bacterial adhesion on hydro-softened chitosan thin films, we developed a stochastic simulation framework grounded in interfacial contact mechanics. Adhesive contact was assessed by coupling the Johnson–Kendall–Roberts (JKR) model^56^ with Griffith energy balance criterion,^57^ yielding a substrate-specific threshold for stable attachment. Interaction energies between bacteria and substrate were calculated using an extended Derjaguin–Landau–Verwey–Overbeek (xDLVO) model.^58,59^ The stochastically computed interaction energies were compared against the JKR-Griffith threshold at each instance to simulate discrete adhesion events. To validate these outcomes, hydro-softened and unsoftened chitosan films were incubated *in vitro*. Subsequently, adhesion outcomes were quantified through Scanning Electron Microscopy (SEM) image-based morphological analysis.

From our adhesion threshold model (see SI Notes), we find that the threshold energy required to form a stable adhesive interface exhibits nonlinear scaling with both the effective elastic modulus (E*) and the interfacial energy (Δγ). Specifically, the threshold follows a −2/3 power law with respect to E*, and a 5/3 power law with respect to Δγ:

1 $$a_{\mathrm{critical}}=\sqrt[{3}]{\frac{9\pi \Delta \gamma R^{2}}{2E^{*}}}$$

where *a*_critical_ is the critical contact radius and *R* is the effective contact radius.

Hydro-softening is driven by the localized entrapment of interfacial water within the amine-hydroxyl-rich free volume domains of the chitosan.^54^ Because this confined water is distributed periodically throughout the film, the resultant softening occurs not only at the macroscopic scale but also at the cellular level. We hypothesized that this microscale softening would enable nonlinear scaling effects (eq 1) with E* and Δγ to persist in governing cellular-level interactions.

To visualize the effect of elastic modulus on adhesion outcomes, a dimensionless adhesion threshold was plotted against substrate stiffness, normalized to the hydro-softened baseline of 1.8 MPa (Figure 2A). The unsoftened regime (85 MPa) occupied a markedly higher energy barrier zone, suggesting that increased rigidity dramatically suppresses adhesion. The area under this curve can be interpreted as a proxy for the cumulative likelihood of successful bacterial attachment, offering a theoretical basis to predict total adhered counts across chitosan thin films of differing stiffness (hydro-softened and unsoftened). This inverse trend highlights the critical relationship between bacterial attachment to substrate mechanics within the quasistatic regime, where adhesion is predominantly governed by instantaneous energetic compatibility.

**Figure 2:**
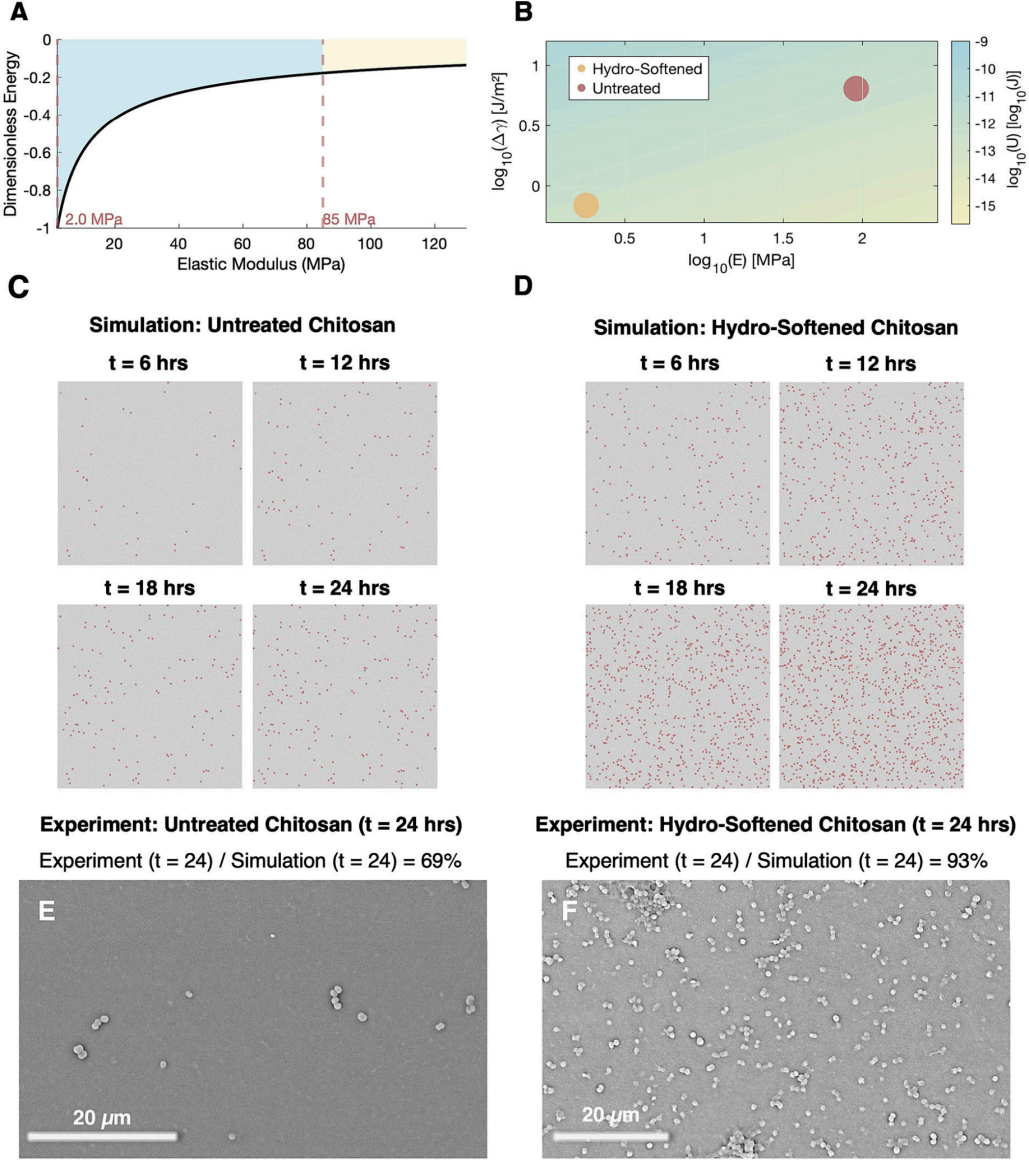
Adhesion governed by elastic modulus and interfacial energetics. (A) Power–law relationship between the adhesion threshold and elastic modulus, favoring softer substrates. (B) Multivariable contour plot illustrating the dependence of adhesion energy on elastic modulus and surface energy. (C) Simulated adhesion over 24 h on unsoftened chitosan. (D) Simulated adhesion over 24 h on hydro-softened chitosan. (E) and (F) Unaltered SEM micrographs of bacterial adhesion at 24 h for unsoftened and hydro-softened, indicating an experimental-simulation agreement of 69% for unsoftened and 93% for hydro-softened.

To contextualize the relationship between elastic moduli of the chitosan films and adhesion behavior, a logarithmic contour plot was generated based on the combined threshold relations (Figure 2B), where the energetic threshold, *U*_threshold_, was mapped as a power law function, dependent on E* and Δγ. The contour reflects the predicted magnitude of log *U*_threshold_, with the lightly colored regions indicating a lower energy barrier and stronger adhesion propensity. The data points represent the hydro-softened and unsoftened chitosan films, positioned according to their measured elastic modulus and Δγ from simulated calibrations. The hydro-softened film occupies a region having a substantially lower modulus and reduced surface energy, falling within a domain of enhanced adhesion probability, highlighting the coupled effect of simultaneous reductions in both parameters. In contrast, the unsoftened chitosan film lies in a higher-threshold region, where the combination of high stiffness and elevated surface energy leads to a reduced likelihood of successful bacterial adhesion. On hydro-softened chitosan thin films, the elastic modulus was reduced from 85 to 1.8 MPa (see SI, Figure S1) and the interfacial energy was decreased from 6.4 to 1.5 J/m^2^. Collectively, the adhesion threshold energy was reduced from −1.6 × 10^–12^ J on the unsoftened surface to −1.2 × 10^–12^ J on the hydro-softened substrate, enhancing the probability of adhesion across the interface. This predictive framework was subsequently validated with a stochastic adhesion simulation corroborated by *in vitro* studies.

To further validate this theoretical framework, we implemented a stochastic simulation model, where each adhesion outcome was modeled as a binary event – either successful attachment or unsuccessful – based on a comparison between a stochastically sampled xDLVO energy (see SI, eqs S1–S4) and a substrate-specific adhesion threshold.

Subsequently, these threshold shifts were encoded into the simulation model. If adhesion were to occur at each instance, a bacterium was seeded on a binary grid, and the collective adhesion status was evaluated at four incubation time points (6, 12, 18, and 24 h), implementing a logistic growth function in estimating the bacterial population size at each incubation time point. Compared to the unsoftened chitosan thin film (Figure 2C), the hydro-softened chitosan thin film (Figure 2D) consistently produced a visible increase in total adhered bacteria compared to the unsoftened substrate. These outputs aligned closely with experimental results, comparing at an accuracy rate of 69% (*in vitro*) for unsoftened and 93% for hydro-softened chitosan films. The discrepancy in the accuracy may stem from the inability of the model to fully capture the complex, nonlinear coupling between the elastic modulus and interfacial energy, a relationship that likely becomes more pronounced under stiffer, less-hydrated conditions.

SEM images were processed to quantify bacterial attachment on unsoftened (Figure 2E) and hydro-softened chitosan films (Figure 2F) postincubation. Unsoftened chitosan thin films exhibited sparse adhesion distribution, whereas hydro-softened chitosan thin films showed markedly greater surface coverage. These differences in adhesion outcomes for the unsoftened and the hydro-softened chitosan thin films are consistent with theoretical and simulated predictions, offering visual and empirical confirmation of enhanced adherence on hydro-softened surfaces. Increased adhesion in hydro-softened thin films were also primarily observed through single cell attachment rather than localized colonization.

Bacterial adhesion was quantified through a multistep image analysis pipeline (see SI, Figure S2A–F), designed to resolve both single-cell and aggregated adhesion outcomes. SEM images were preprocessed using adaptive histogram equalization to enhance local contrast, followed by Gaussian filtering and background correction to minimize artifacts. Adaptive thresholding and area-based filtering isolated bacterial regions from the background. Subsequently, watershed segmentation and distance transformation delineated overlapping cells within clusters, enabling connected-component analysis and the extraction of morphological features. Bacteria were then classified as either single cells or aggregates based on these shape metrics, providing a morphology-resolved quantification of adhesion. Statistical comparisons of simulated and experimental adhesion between unsoftened and hydro-softened films were performed with two-tailed independent *t* tests. Two-way analysis of variance (ANOVA) was led with treatment (hydro-softened, unsoftened) and morphology (single, aggregated) as independent factors to evaluate the significance of morphological shifts in bacterial adhesion.

The simulations yielded consistently higher bacterial adhesion on hydro-softened substrates, with a mean of approximately 1000 adhered particles compared to 180 on unsoftened films (Figure 3A) (*p* < 0.0001). To enable a direct comparison with experimental outcomes, SEM-derived bacterial counts were scaled for the mean area occupied per bacterium (∼0.0077% of the surface). These experimental results showed that hydro-softened films supported an average of 990 total adhered bacteria (*p* < 0.0001), while unsoftened films averaged 180, aligning closely with simulated predictions (Figure 3B). Additionally, the connected-component analysis revealed a marked increase in single-cell adhesion on hydro-softened films compared to unsoftened films (Figure 3C,D). Two-way ANOVA showed a significant main effect of treatment (*p* ≈ 0.005) and a marginal interaction between treatment and adhesion morphology (*p* ≈ 0.078), suggesting that hydro-softening not only increases overall adhesion, but may also promote a shift toward a more disperse, individual attachment, consistent with uniform interfacial engagement. Replicates of the images and counts can be found in SI, Data.

**Figure 3:**
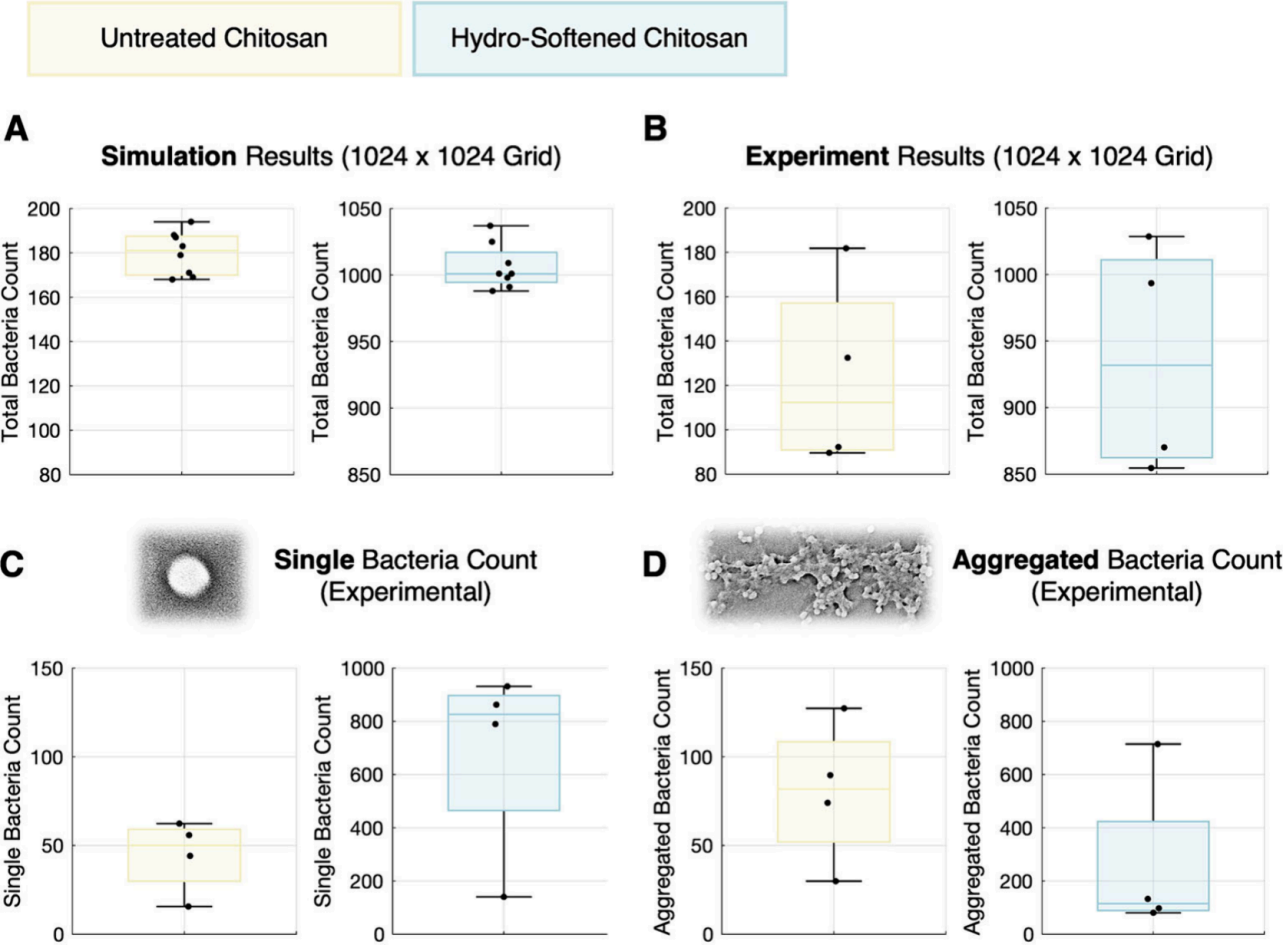
Quantification of simulated and experimental adhesion. (A) Simulation results of total adhered bacteria count after 24 h on unsoftened (left) and hydro-softened (right) chitosan thin films (1024 × 1024 grid; *n* = 8). (B) Corresponding experimental bacterial adhesion counts from SEM analysis (*n* = 4). (C) Experimentally measured counts of isolated single bacteria to unsoftened and hydro-softened films, demonstrating preferential adhesion enhancement (*n* = 4). (D) Aggregated bacterial counts on both films, determined experimentally, highlighting a shift toward predominantly single bacterial attachment on hydro-softened chitosan (*n* = 4).

Previous studies have revealed that stiffness-mediated bacterial adhesion is not a universal trend, but rather an intricate material-dependent relationship influenced by surface chemistry and polymer architecture. Straub et al.^60^ reports that adhesion increased on softer polydimethylsiloxane when the surface remained chemically inert. Similarly, Guégan et al.^61^ reports increased Gram-positive bacterial adhesion on agarose hydrogels when they were softened. In both studies, stiffness was modulated by varying the cross-linking density, which inherently altered the underlying polymer network.

Our work supplements these previous works with hydro-softened chitosan, where mechanical softness is introduced via interfacial water confinement. Despite the presence of strong bacteria-amine interactions, we find that softening alone significantly alters adhesion outcomes, suggesting that the biological response to stiffness is not solely determined by stiffness, but also the mechanism through which softness is achieved. Hydro-softening offers a distinct pathway for modulating bacterial attachment and invites further mechanistic exploration into the role of mechanics-mediated bacterial adhesion.

This work finds that entropically forced water ordering fundamentally enables hydro-softening-mediated cell adhesion. By inducing angstrom-scale pockets of interfacial water during substrate-mediated condensation, chitosan undergoes global and localized reductions in stiffness and surface energy without any chemical modification. These pseudosolid hydration zones alter the energetic landscape of initial contact, lowering the threshold for phase one bacterial adhesion. In contrast to strategies that rely on biochemical patterning or ligand presentation, hydro-softening operates through physical confinement alone, enabling a passive, tunable, and spatially heterogeneous route to modulate interfacial bioactivity. More broadly, this mechanism reframes mechanical processes not merely as a supporting factor in adhesion, but as an active, designable element for soft material interfaces. This finding opens new opportunities in reshaping our thoughts on how interfacial mechanics can be utilized as a lever to guide cell–material interactions with molecular precision. Finally, hydro-softened chitosan presents a promising platform for developing adhesion-tunable coatings in numerous applications.

## Experimental Section

Tetraethyl orthosilicate (TEOS), medium molecular weight chitosan, and hydrochloric acid (HCl) were purchased from Millipore Sigma. Powdered sodium hydroxide was obtained from Fisher Scientific. The Sylgard 184 silicone elastomer kit was purchased from Dow Inc. via Krayden Inc., and silicon wafers were sourced from MSE Supplies.

One g of medium molecular weight chitosan was dissolved in an aqueous acidic solution of 6.2 mL of 1 M HCl and 100 mL of deionized (DI) water. 260 μL of TEOS was added to achieve a 40% degree of substitution upon hydrolysis, followed by stirring for an additional hour. Polydimethylsiloxane (PDMS) was prepared using the Sylgard 184 elastomer kit. After sequential cleaning of silicon wafers with acetone, isopropanol, and DI water, PDMS was spin-coated at 800 rpm for 60 s, yielding dry films approximately 200 μm thick. PDMS films were then thermally cured at room temperature (in preparation for unsoftened chitosan films) and 160 °C (in preparation for hydro-softened chitosan films) for 48 h to introduce steric conditions, which enables hydro-softening. When PDMS is mechanically stiffened, this exponentially amplifies the water confinement within the polymer, enhancing the local entropic penalty associated with hydration. Postcuring, the PDMS substrates were treated in a UV–O_3_ chamber for 2 h to generate surface hydroxyl and carboxyl functionalities. Hydrolyzed chitosan sol was then spin-coated onto the treated PDMS at 1200 rpm for 60 s. The condensation reaction between the chitosan sol and surface hydroxyls expels the solvent, forming the chitosan films within 25 to 35 min.

*Staphylococcus epidermidis* (Gram-positive) was selected as the model organism for *in vitro* bacterial adhesion assays. Prior to bacterial exposure, hydro-softened and unsoftened chitosan films were mounted on 3 M ScotchBlue tape to isolate one surface, ensuring that only the side of interest was exposed to the bacterial suspension. The films were placed in 6-well culture plates and incubated with 5 mL of bacterial suspension (optical density = 0.3, approximately 5 × 107 cells/mL) prepared in tryptic soy broth (TSB). Incubation was performed at 37 °C for 24 h under static and humidified conditions. Postincubation, samples were rinsed three times with phosphate-buffered saline (PBS) to remove nonadherent bacteria, then transferred into 50 mL tubes containing 5 mL of PBS before SEM imaging.

Samples were immersed in 70% v/v ethanol for 15 min and dehydrated at 40 °C to stabilize the surface for SEM observation. Samples were gold sputtered for 45 s with the Cressington 108 sputter coater to render the surface conductive. Chitosan films were characterized using SEM with a Phenom XL G2 microscope operated at 10 kV. For quantitative image analysis, grayscale SEM images (see SI, Figure S2A) were enhanced with adaptive histogram equalization to improve local contrast while avoiding overenhancement (see SI, Figure S2B). Then, a Gaussian filter was applied to reduce high-frequency noise, followed by background subtraction to correct for illumination nonuniformity (see SI, Figure S2C). The resulting images were binarized using adaptive thresholding and refined through area-based filtering to remove small artifacts. To resolve the aggregation morphology, watershed segmentation and distance transformation were applied (see SI, Figure S2D). The resulting binary masks were smoothened (see SI, Figure S2E) for connected component analysis, to label and quantify the individual particles and extract key morphological descriptors (area, circularity, and solidity) (see SI, Figure S2F).

## Supplementary Material



## Data Availability

In compliance with institutional data management policies, the full codebase is maintained on Georgia Tech’s GitHub Enterprise instance. A stable public version is available at https://github.com/hseo47/ABC and has been archived with a permanent DOI at 10.5281/zenodo.15474840. This public version includes usage instructions, example data sets, and all necessary dependencies for replication.

## Notes

Supporting figures, interfacial energetics and adhesion threshold, scaling parameters, and SEM images (PDF)

## §

A.T. is with the School of Civil and Environmental Engineering, Georgia Institute of Technology, Atlanta, GA, 30332–0405, USA
