# Supplementary material for: Enhancing Bacterial Adhesion with Hydro-Softened Chitosan Films
Source: ACS Macro Lett. 2025 Jul 30;14(8):1155–61. doi: 10.1021/acsmacrolett.5c00374 (PMC12369014; doi:10.1021/acsmacrolett.5c00374)

Supporting Information for Enhancing Bacterial Adhesion with Hydro-Softened Chitosan Films

Hojin Seo[†], Xiaoqing Yu[†], Anuja Tripathi[‡, §], Julie Champion[‡], and Tequila A. L. Harris[†, *]

[†] George W. Woodruff School of Mechanical Engineering, Georgia Institute of Technology, Atlanta, GA, 30332-0405, USA

[‡] School of Chemical and Biomolecular Engineering, Georgia Institute of Technology, Atlanta, GA, 30332-0405, USA

[§] A. T. is presently with the School of Civil and Environmental Engineering, Georgia Institute of Technology, Atlanta, GA, 30332-0405, USA.

*Corresponding author: tharris3@gatech.edu

Hydro-Softening; Flexible Thin Films; Chitosan; Bio-adhesion; S. epidermidis

SUPPORTING INFORMATION FIGURES

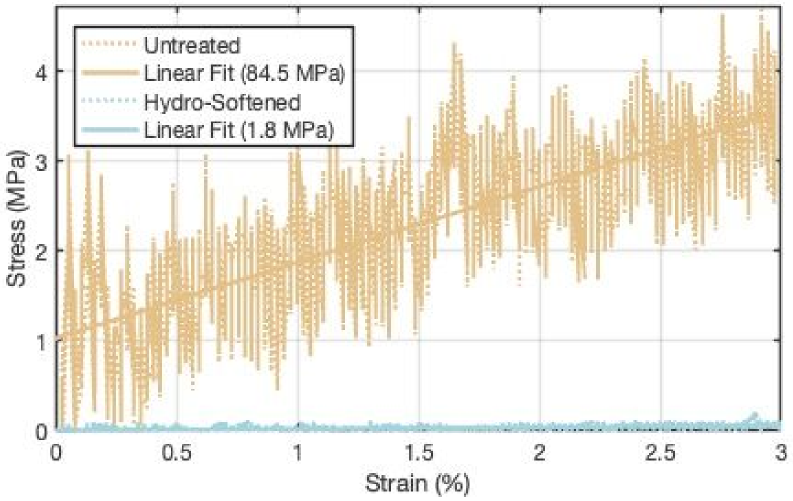

Figure S1. Stress-strain behavior of unsoftened and hydro-softened films. Stress-strain responses of unsoftened and hydro-softened films shown over the first 3% strain, where linear fits to the elastic regions yield elastic moduli of 84.5 MPa and 1.8 MPa for the unsoftened and hydro-softened films, respectively.

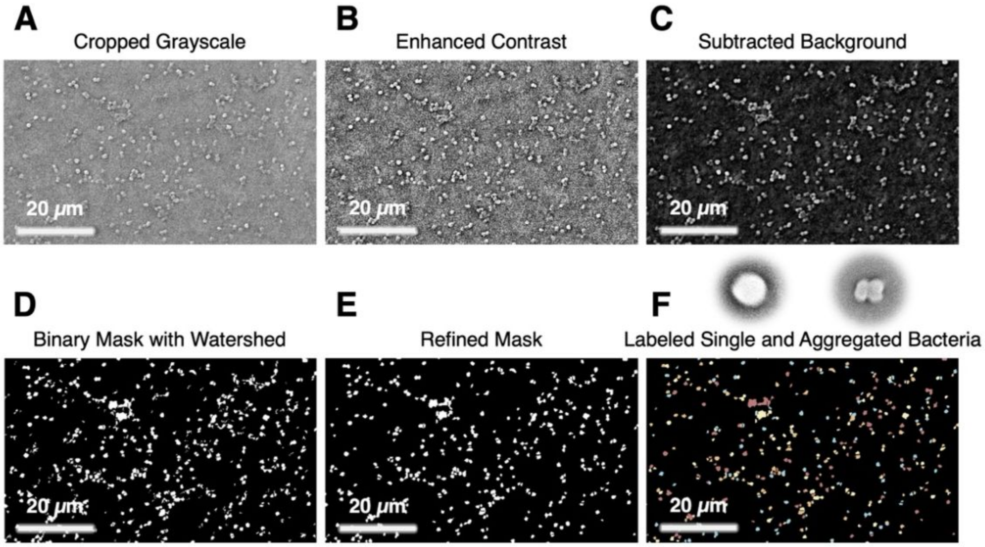

Figure S2. Image processing workflow for adhesion quantification. (A) Cropped grayscale input from SEM imaging of adhered bacteria on hydro-softened chitosan. **(B)** Enhanced contrast applied to improve edge definition and background differentiation. **(C)** Background subtraction to isolate high-contrast features. **(D)** Binary mask with thresholding and watershed segmentation to distinguish individual particles. **(E)** Refined mask after morphological filtering and noise removal. **(F)** Final segmentation and labelling of single and aggregated bacteria based on features and proximity.

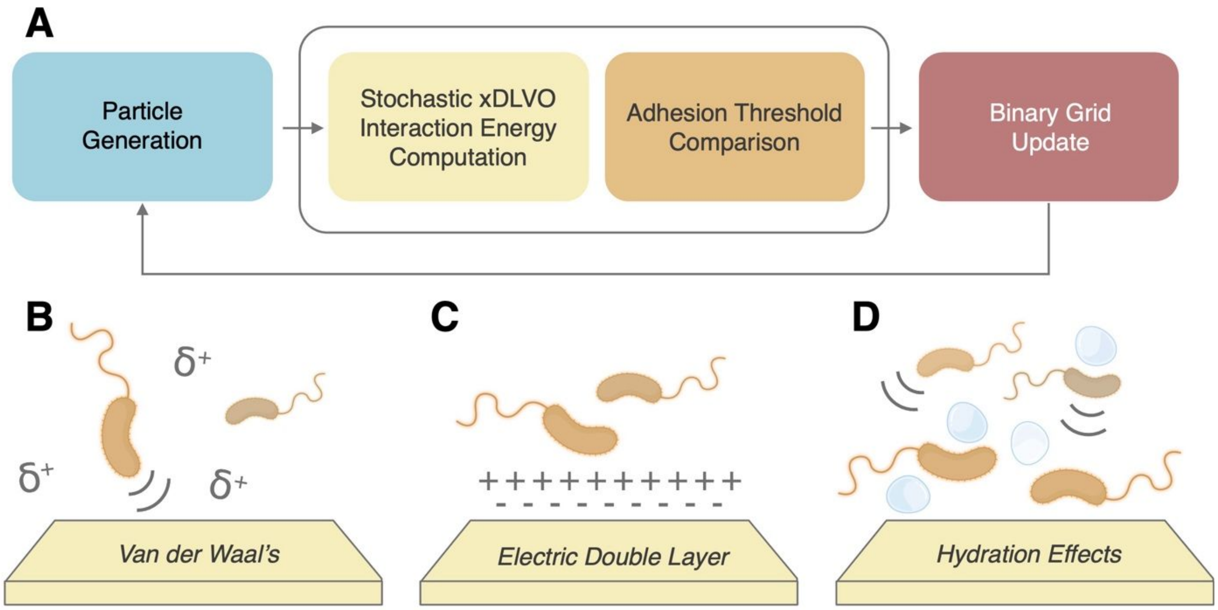

Figure S3. Simulation framework and components of xDLVO energy. (A) Simulation loop schematic. **(B)** Van der Waals attraction as dipole-induced interactions between bacteria and substrate interfaces. **(C)** Electrostatic repulsion arising from electric double layer interactions. **(D)** Hydration-mediated repulsion driven by confined interfacial water.

SUPPORTING INFORMATION NOTES

INTERFACIAL ENERGETICS AND ADHESION THRESHOLD

Kinetic Monte Carlo Stochastic Simulation

To quantitatively model bacterial adhesion dynamics on hydro-softened chitosan films, we developed a stochastic simulation framework based on the kinetic Monte Carlo (kMC) method (Figure S3A). The simulation captures bacterial adhesion as a time-dependent probabilistic process governed by interfacial energy barriers and mechanical properties of the film. Bacterial population growth over time was modelled using a logistic growth function, defined as:

$$N(\mathrm{t}) = \frac{K}{1+\left(\frac{K-N_0}{N_0}\right)e^{-\mathrm{rt}}}.$$

Supplemental Table 1. Summary of parameters used in the logistic growth model for simulating time-resolved bacterial population dynamics in the kMC framework.

N(t)	*Bacterial population at time t*	Eq. 1	-
N_0	*Initial population*	0.3	- (Optical Density)
K	*Carrying capacity*	2.0	- (Optical Density)
r	*Bacterial growth rate*	0.4	hr^{-1}
t	*Simulation time points*	6, 12, 18, 24	hr

At each time point, the number of simulated bacteria was computed using the logistic growth model (Eq. S1) and scaled to a maximum of 1000 particles at 24 hours. This dynamic population represents the evolving bacterial load and enables probabilistic evaluation of adhesion events over time, allowing the simulation to capture not only spatial variation across the surface but also changes in colonization pressure. Then, bacteria were distributed across a binary grid to simulate unbiased interactions.

Adhesion was modelled as a binary outcome – either successful attachment or rejection – based on a comparison between the computed xDLVO interaction energy and a substrate-specific energy threshold derived from contact mechanics (Table 1). If the interaction energy exceeded the threshold, the bacterium was considered adhered.

xDLVO Interaction Model

To compute the interaction energy between bacteria and hydro-softened chitosan films, we developed an extended DLVO (xDLVO) model (Table 2) incorporating classical van der Waals (Figure S3B) and electrostatic interactions (Figure S3C), along with short-range hydration forces (Figure S3D). While traditional DLVO theory omits solvent structuring at the interface, these effects become significant in hydrated polymer systems, where water is not merely a medium but a structural component of the material. To account for this, an exponentially decaying short-range hydration term was added to the total interaction energy, evaluated at a fixed separation distance. The xDLVO model states:

$$U_{\mathrm{xDLVO}}(\mathrm{r}) = U_{\mathrm{vdW}}(\mathrm{r}) + U_{\mathrm{edl}}(\mathrm{r}) + U_{\mathrm{hydration}}(\mathrm{r}), \qquad \text{Eq. S1}$$

Supplemental Table 2. Summary of interaction energy equations used in the xDLVO model. Each component represents a distinct physical contribution to the total interaction energy: long-range van der Waals attraction, electrostatic double-layer repulsion, and short-range hydration repulsion.

$U_{\mathrm{vdW}}(\mathrm{r})$	*Van der Waals*	$-\frac{A_H}{6\mathrm{r}}$	Eq. S2
$U_{\mathrm{edl}}(\mathrm{r})$	*Electrostatic double layer*	$\frac{64\varepsilon_0\varepsilon_r k_B T}{\varkappa}\tanh^2(\frac{e\psi_0}{4k_B T})e^{-\varkappa\mathrm{r}}$	Eq. S3
$U_{\mathrm{hydration}}(\mathrm{r})$	*Hydration*	$H_0 e^{-\mathrm{r}/\lambda}$	Eq. S4

For each bacterium in the simulation, a unique set of constants (Table 3) and physical parameters was stochastically sampled from defined ranges (Table 4), and the corresponding xDLVO interaction energy was calculated. This energy was then compared against a substrate-specific adhesion threshold derived from the Griffith-JKR framework, which was -1.6×10^{-12} J for unsoftened chitosan films and -1.2×10^{-12} J for hydro-softened films. Adhesion was recorded when the computed energy fell below this threshold, representing successful contact formation. This formulation integrates interfacial mechanics and hydration-mediated effects to capture quasi-static attachment behavior. The derivation of the energy threshold is detailed in the following subsection. This comparison governs the probabilistic outcome and forms the core decision-making within the kMC simulation.

Supplemental Table 3. Fixed physical and environmental parameters used in xDLVO calculations.

ε_0	*Vacuum permittivity*	8.854×10^{-12}	Fm^{-1}
ε_r	*Relative permittivity of water*	78.5	Fm^{-1}
k_B	*Boltzmann's constant*	1.3806×10^{-23}	JK^{-1}
T	*Temperature*	298	K
e	*Elementary charge*	1.602×10^{-19}	C
N_A	*Avogadro's number*	6.022×10^{23}	mol^{-1}
λ	*Characteristic decay length*	1	nm

Supplemental Table 4. Stochastically sampled variables (xDLVO).

A_H	*Hamaker constant*	$0.5 \times 10^{-20} \sim 5 \times 10^{-20}$	J
ψ_0	*Surface potential*	$10 \sim 50$	mV
$\varkappa$	*Debye length (function of I)*	$\sqrt{\frac{2N_A e^2 I}{\varepsilon_0 \varepsilon_r k_B T}}$	m
I	*Ionic strength*	$10 \sim 100$	mM
H_0	*Hydration energy coefficient*	$1 \times 10^{-20} \sim 1 \times 10^{-19}$	J

Adhesion Threshold Model

To determine whether adhesive contact is energetically favorable, we implemented an energy-based criterion derived from the Johnson–Kendall–Roberts (JKR) contact mechanics framework. Bacteria are assumed to adhere when its xDLVO interaction energy falls below a substrate-specific threshold, representing a quasi-static balance between the elastic cost of deformation and the interfacial energy gained through contact. This threshold is defined by incorporating the Griffith energy criterion, which equates the energy required to create new surfaces with the elastic energy released during separation. In our context, this principle is adapted to model the initiation of contact: adhesion occurs when the energy gained by forming an interface exceeds the mechanical resistance to deformation. Treating bacterial attachment as a quasi-static event allows this fracture-based framework to define a stable attachment threshold (Table 5). This threshold energy is integrated into the kMC simulation as the probabilistic cutoff that governs whether each sampled bacterium is accepted or rejected at the point of contact, linking microscale mechanics to population-level adhesion outcomes. The bacterium is

approximated as a rigid sphere in contact with a flat substrate, and the total potential energy of interaction derives the adhesion criterion applied in the stochastic simulation:

$$U_{\text{total}}(a) = U_{\text{elastic}}(a) - U_{\text{surface}}(a) = \frac{E^{*}a^{5}}{15R^{2}} - \pi a^{2}\Delta\gamma.$$

The system reaches equilibrium when the energy release rate, G, equals the interfacial work of adhesion, $\Delta\gamma$. In this framework, G is the rate at which stored energy is released as the contact area increases, and is defined as the derivative of the total energy with respect to the contact area, where $A = \pi a^2$:

$$G = -\frac{dU_{\text{total}}}{dA} = \frac{E^{*}a^{3}}{6\pi R^{2}} + \Delta\gamma.$$

At the critical contact radius, a_{critical}, where $G = \Delta\gamma$, we can obtain the following closed-form Eq. 1.

This model defines a substrate-specific energy threshold for stable adhesion. The expression scales nonlinearly with the material parameters, following a -2/3 power law with respect to the effective modulus E^* and a 5/3 power law with respect to the interfacial energy $\Delta\gamma$. These thresholds – computed separately for unsoftened and hydro-softened chitosan films – are used within the kMC framework to determine whether bacterial contact results in adhesion. This formulation highlights that surface mechanical properties, even in the absence of chemical modification, can influence bacterial adhesion by modulating the energy landscape at the interface.

Supplemental Table 5. Mechanical parameters used in the JKR-based adhesion model.

E^*	*Effective elastic modulus*	$\frac{1}{E^*} = \frac{1 - \nu^2_{\text{chitosan}}}{E_{\text{chitosan}}} + \frac{1 - \nu^2_{\text{bacteria}}}{E_{\text{bacteria}}}$	MPa
a	*Contact radius*	$\sqrt[3]{\frac{9\pi\Delta\gamma R^2}{2E^*}}$	μm
R	*Effective contact radius*	1	μm
$\Delta\gamma$	*Effective work of adhesion*	-1.6×10^{-12}; -3.5×10^{-13}	Jm^{-2}

SUPPORTING INFORMATION DATA

Scaling Parameters and SEM Images

One bacteria occupies 0.0077% of the grid

Image Analyzed: unsoftened1.jpg
Number of detected bacteria: 36
Total surface coverage by bacteria: 1.40%
Single-bacteria coverage: 0.43% of total image
Aggregated-bacteria coverage: 0.98% of total image
(Within bacterial area: 30.59% single, 69.41% aggregate)

Image Analyzed: unsoftened2.jpg
Number of detected bacteria: 32
Total surface coverage by bacteria: 1.02%
Single-bacteria coverage: 0.34% of total image
Aggregated-bacteria coverage: 0.69% of total image
(Within bacterial area: 32.93% single, 67.07% aggregate)

Image Analyzed: unsoftened3.jpg
Number of detected bacteria: 26
Total surface coverage by bacteria: 0.71%
Single-bacteria coverage: 0.48% of total image
Aggregated-bacteria coverage: 0.23% of total image
(Within bacterial area: 67.37% single, 32.63% aggregate)

Image Analyzed: unsoftened4.jpg
Number of detected bacteria: 14
Total surface coverage by bacteria: 0.69%
Single-bacteria coverage: 0.12% of total image
Aggregated-bacteria coverage: 0.57% of total image
(Within bacterial area: 17.97% single, 82.03% aggregate)

Image Analyzed: softened1.jpg
Number of detected bacteria: 514
Total surface coverage by bacteria: 7.92%
Single-bacteria coverage: 7.17% of total image
Aggregated-bacteria coverage: 0.75% of total image
(Within bacterial area: 90.48% single, 9.52% aggregate)

Image Analyzed: softened2.jpg
Number of detected bacteria: 476
Total surface coverage by bacteria: 7.65%
Single-bacteria coverage: 6.64% of total image
Aggregated-bacteria coverage: 1.02% of total image
(Within bacterial area: 86.74% single, 13.26% aggregate)

Image Analyzed: softened3.jpg
Number of detected bacteria: 466
Total surface coverage by bacteria: 6.70%
Single-bacteria coverage: 6.08% of total image
Aggregated-bacteria coverage: 0.62% of total image
(Within bacterial area: 90.68% single, 9.32% aggregate)

Image Analyzed: softened4.jpg
Number of detected bacteria: 142
Total surface coverage by bacteria: 6.58%
Single-bacteria coverage: 1.08% of total image
Aggregated-bacteria coverage: 5.50% of total image
(Within bacterial area: 16.35% single, 83.65% aggregate)

Scaling Parameters for Processed SEM Images

Index	Rigid	Soft
1	187	988
2	194	1001
3	188	1037
4	171	998
5	183	1009
6	169	991
7	168	1025
8	179	1001

unsoftened1.jpg

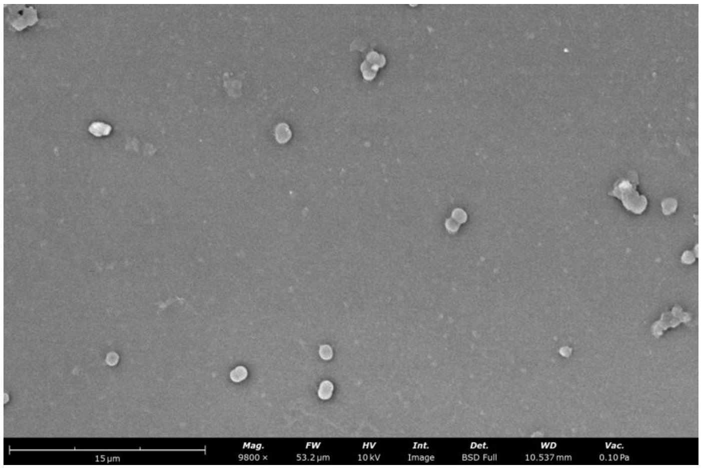

unsoftened2.jpg

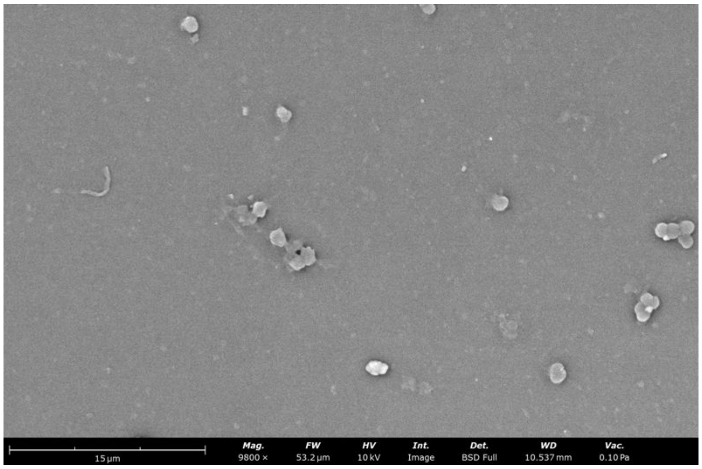

unsoftened3.jpg

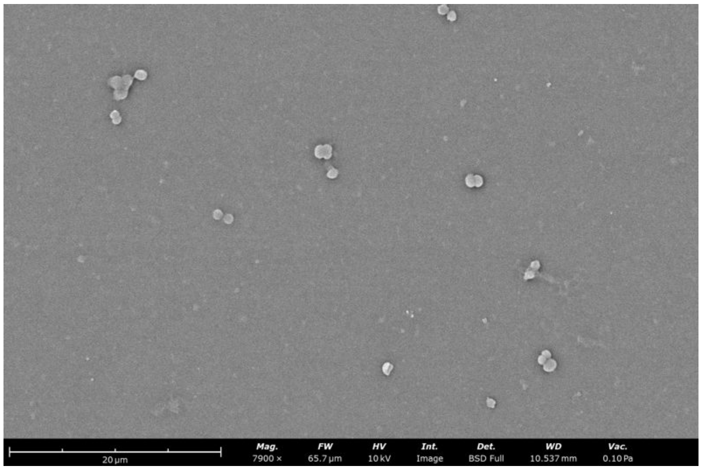

unsoftened4.jpg

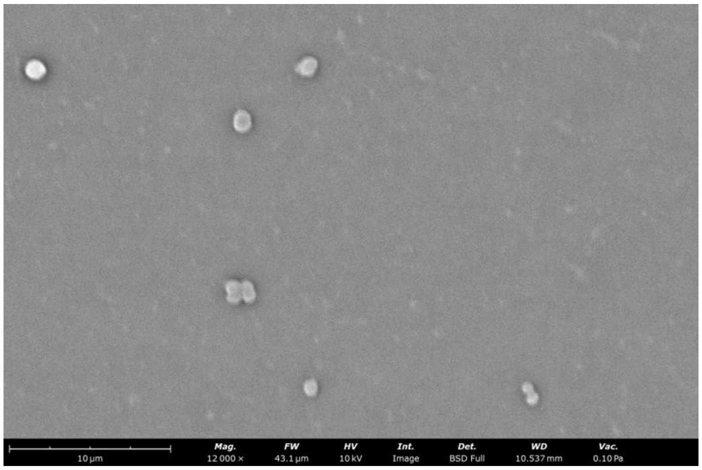

softened1.jpg

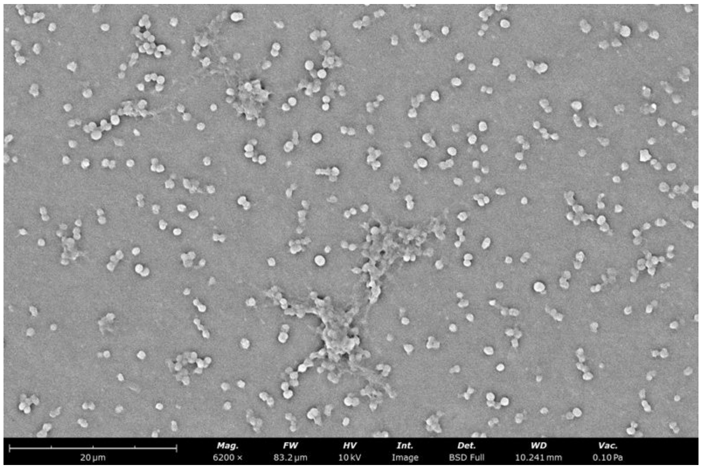

softened2.jpg

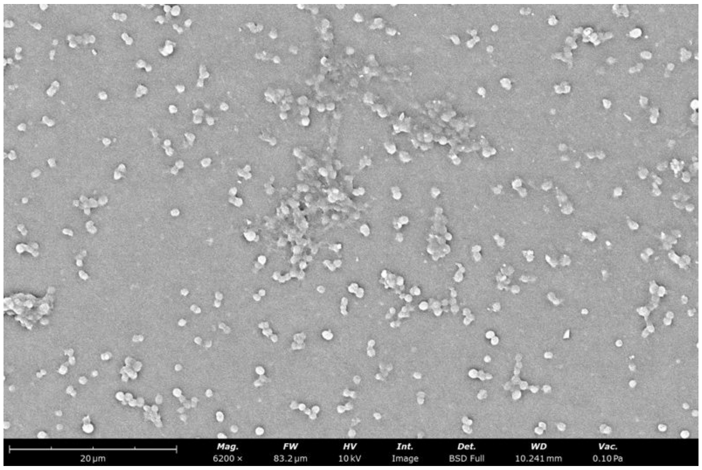

softened3.jpg

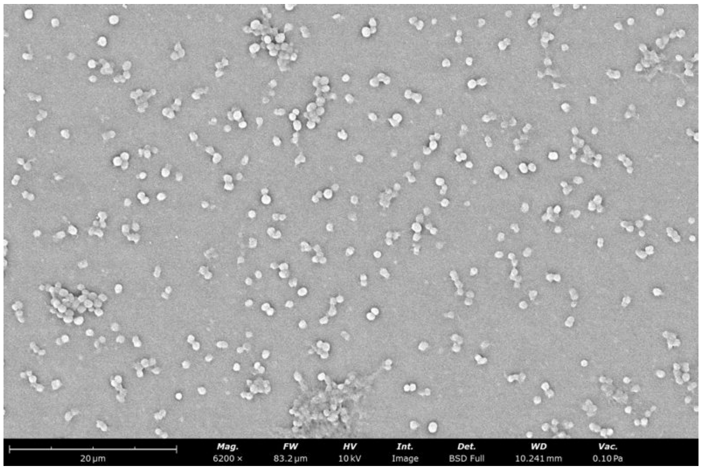

softened4.jpg

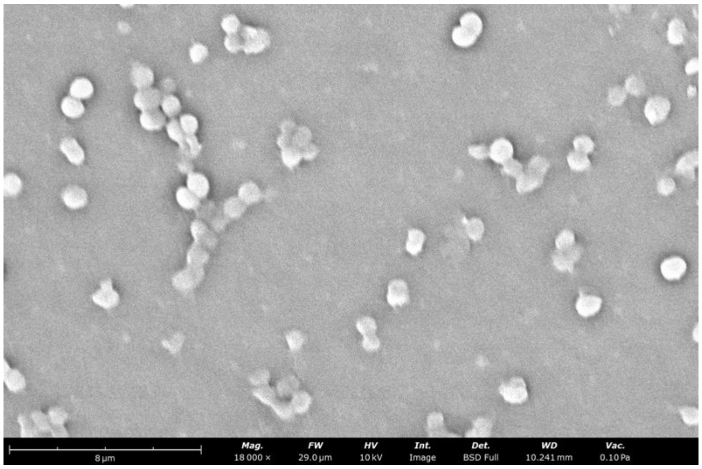

Supplement: Supplementary file 1 [file mz5c00374_si_001.pdf]
